# Editorial: Equity in health: placing human diversity at the heart of neuroscientific research

**DOI:** 10.3389/fnins.2025.1629508

**Published:** 2025-06-13

**Authors:** Sivaniya Subramaniapillai, Aurelie de Rus Jacquet

**Affiliations:** ^1^Centre for Research in Neurosciences, Department of Clinical Neurosciences, Lausanne University Hospital (CHUV) and University of Lausanne, Lausanne, Switzerland; ^2^Centre de recherche du CHU de Québec, Québec, QC, Canada; ^3^Département de Psychiatrie and Neurosciences, Université Laval, Québec, QC, Canada

**Keywords:** health equity, determinants of health, intersectionality, diversity in neuroscience, neurology

Medical research that meaningfully serves global populations must reflect the complexity of human diversity. However, historically, much of biomedical and public health research has failed to incorporate key dimensions such as sex/gender, ethnicity, culture, and socioeconomic status (Braveman and Gottlieb, 2014; George et al., 2014; Oh et al., 2015; Subramaniapillai et al., 2024). Health outcomes—particularly those related to brain and neurological function—are shaped not only by biological factors but also by environmental factors and structural inequities that influence access to care, exposure to risk, and inclusion in scientific research (National Academies of Sciences, 2023; Legaz et al., 2025; Williams and Collins, 2013). This Research Topic brings together five timely contributions that illuminate how social, economic, and demographic disparities intersect to shape major neurological and public health challenges—from stroke and concussion to gender-based violence and premature mortality. Together, these contributions underscore the urgent need for equity-driven approaches in both research and policy, offering insight into the systemic reforms needed to close persistent gaps in health outcomes.

One major theme emerging from these studies is the influence of social and geographic disparities on neurological outcomes. Using global data spanning three decades, Liu et al. examined the burden of ischemic stroke among adults aged 20 years and older from 1990 to 2021. By incorporating subnational geographic divisions in addition to national stratification, the authors revealed how changes in age-standardized mortality and disability-adjusted life years (DALYs) are linked to the sociodemographic index, a composite marker of income, education, and fertility rate. These findings underscore the need for locally tailored health policy interventions that reflect both regional and population-specific needs.

Such disparities are not limited to the global scale. In the United States, stark health gaps exist among population subgroups even within the same geographic regions. Nadeau et al. investigated this issue by analyzing premature mortality among American Indians in North Dakota, who experience some of the highest age-adjusted mortality rates in the country. Their analysis further support previous observations of deep-rooted health inequities (Carron, 2020). By stratifying the data by age group and sex, the authors highlighted the social determinants underlying these elevated mortality rates and proposed community-informed, culturally sensitive solutions ranging from prevention and outreach to institutional collaborations designed to close persistent care gaps.

The consequences of overlooking diversity are also evident in how research itself is conducted. MacEachern et al. examined whether concussion literature accurately represents Black/African American individuals, who are overrepresented in high-risk sports but often underdiagnosed. Their meta-analysis revealed striking inconsistencies across data sources—hospital records, national surveys, and sports clinics—with concussion rates varying depending on the setting. These inconsistencies raise concerns about how structural bias, whether through study design, access to care, or diagnostic criteria, can distort our understanding of injury risk and outcomes, ultimately limiting the relevance of care delivery.

In parallel, a gendered lens on brain health reveals how sex and socioeconomic context interact to influence neurological risk and vulnerability. Ronne-Engström and Friberg examined the socioeconomic profiles of 890 Swedish patients with spontaneous subarachnoid hemorrhage, and compared them to matched controls. Living in sparsely populated areas emerged as a key risk factor, but striking sex differences were also evident. Women with subarachnoid hemorrhage were more economically vulnerable—often being unemployed, single, and having a lower income—while men showed a different type of vulnerability related to changes in civil status. These findings suggest the need for targeted, preventative healthcare strategies that consider socioeconomic context, particularly in rural and marginalized populations.

This need for intersectional, context-sensitive healthcare was brought into sharper focus by Taiebine's opinion article, which draws attention to the neurological and psychological consequences of gender-based violence on refugee and migrant women. Despite global action plans that recognize gender-based violence as a public health issue (e.g., World Health Organization, 2016), the neurological consequences of trauma, especially in contexts of forced displacement and limited access to care, remain underexamined. Taiebine argues for the development of integrated, trauma-informed, and culturally sensitive interventions that address the full scope of survivors' needs across health, legal, and social systems.

This Research Topic contributes to an evolving body of evidence that calls for intersectional and community-engaged approaches in neuroscience research and public health policy. These articles show that structural determinants are integral to understanding brain and health outcomes. More importantly, they demonstrate that addressing health inequities requires more than diverse data collection; it requires a transformation in how public health systems identify risk, allocate resources, and design inclusive interventions. However, if research is to serve all people, equity must also extend to who gets to participate in shaping the science itself. Open-access fees, publishing barriers, and structural biases in academia disproportionately affect researchers from low-resource settings and historically marginalized communities (Byrne, 2024; Frank et al., 2023; Kwon, 2022). Without addressing these limitations, we risk perpetuating the very exclusions we seek to correct. As editors and contributors, we believe that equity in research must also include equity in access and authorship.

Moving forward, we encourage the continued integration of intersectional frameworks, culturally grounded definitions of health, and inclusive research methods spanning neuroscience, genomics, epidemiology, and implementation science. By doing so, we can build a research ecosystem in which diversity is not an afterthought but a guiding principle, thereby advancing both the science and the equity it seeks to serve.
